# Comprehensive clinical analysis of gastric-type endocervical adenocarcinoma: a real-world multicenter study

**DOI:** 10.1080/07853890.2025.2584735

**Published:** 2025-11-14

**Authors:** Lele Chang, Yaxin Kang, Yanhong Zhuo, Jing Liu, Lele Zang, Li Li, Ling Lin, Chengzhi Jiang, Huaiwu Lu, Qin Xu

**Affiliations:** ^a^ NHC Key Laboratory of Cancer Metabolism, Department of Gynecology, Fujian Cancer Hospital, Clinical Oncology School of Fujian Medical University, Fuzhou, China; ^b^ Department of Radiation Oncology, Zhangzhou Hospital Affiliated to Fujian Medical University, Zhangzhou, China; ^c^ Clinical Oncology School, Fujian Medical University, Fuzhou, China; ^d^ Department of Gastrointestinal Medical Oncology, Harbin Medical University Cancer Hospital, Harbin, China; ^e^ Department of Gynecologic Oncology, Sun Yat-sen Memorial Hospital, Guangzhou, China; ^f^ Fujian Key Laboratory of Advanced Technology for Cancer Screening and Early Diagnosis, Fujian Cancer Hospital, Fuzhou, China

**Keywords:** Gastric-type endocervical adenocarcinoma, real-world study, clinical characteristics, prognosis

## Abstract

**Background:**

Gastric-type endocervical adenocarcinoma (G-EAC) is a rare malignancy, and its clinicopathological characteristics remain poorly defined. This study aimed to evaluate the real-world features, treatment patterns, and outcomes of patients with G-EAC.

**Methods:**

Clinical data from 124 patients diagnosed with G-EAC between 2012 and 2024 across four tertiary hospitals in China were retrospectively analyzed. Clinicopathological features, therapeutic approaches, and survival outcomes were assessed. Overall survival (OS) was the primary endpoint. Kaplan–Meier and Cox regression analyses were performed to identify prognostic factors.

**Results:**

The median diagnostic age was 55 years (range, 33–82). At presentation, 62.1% of patients had invasion or metastasis, most commonly lymphovascular (47.6%). Surgery was performed in 81.5% of cases, and 84.7% received chemotherapy, primarily platinum-based (81.5%). Radiotherapy was administered to 69.4%. The 1-, 3-, and 5-year OS rates were 78.6%, 54.8%, and 46.1%, respectively. Older age (≥65 years; HR, 4.71; 95% CI, 1.52–14.58; *p* = 0.0071) and advanced stage (*p* < 0.05) predicted poorer OS, while surgery (HR, 0.10; 95% CI, 0.02–0.43; *p* = 0.0019) and multiple chemotherapy cycles (p < 0.05) were independent protective factors.

**Conclusions:**

G-EAC exhibits aggressive behavior and unfavorable prognosis, with a 5-year OS of 46.1%. Multimodal treatment, particularly surgery combined with chemotherapy, remains the cornerstone of management and may improve survival. Prospective multicenter studies are warranted to further define optimal therapeutic strategies for this rare entity.

## Background

As the fourth most common cancer worldwide, cervical cancer accounted for 661,021 new cases and 348,189 deaths in 2022 [1]. Although a broad decline in cervical cancer incidence rates has been observed in most areas of the world over the last few decades with the increased uptake of human papillomavirus (HPV) vaccination, non-HPV-associated cervical cancers, especially adenocarcinoma, account for an increasing proportion of new diagnoses [2]. Gastric-type endocervical adenocarcinoma (G-EAC), recognized as the most prevalent form of non-HPV-related cervical cancer, accounts for 10% of all newly diagnosed cervical adenocarcinomas in Europe and North America, 16% in China and 25% in Japan. This positions G-EAC as the second most common primary adenocarcinoma of the cervix, following usual-type cervical adenocarcinoma (UEA) associated with high-risk HPV infection [3].

The term G-EAC was first described by Kojima et al. in 2007 [4]. The World Health Organization classified it as a distinct type of adenocarcinoma under the category of mucinous carcinoma of the uterine cervix in 2014. The 2020 revised classification classifies it as an independent subtype of non-HPV-related cervical adenocarcinoma [5]. G-EAC is highly aggressive and prone to distant metastases [6]. Most initially diagnosed patients have widespread involvement and are in the advanced stage [7]. Most current treatments for cervical cancer are based on the etiological mechanisms of HPV infection; G-EAC is not related to HPV infection and has an unclear mechanism, thus there are no definitive treatment options for G-EAC [4]. Furthermore, G-EAC is poorly sensitive to chemotherapy and easily resistant to it [4]. High invasiveness, the low preoperative diagnosis rate, the high rate of missed diagnosis and misdiagnosis, and uncertain treatment methods seriously affect the prognosis of patients with G-EAC. Previous studies have found that patients with G-EAC had poor survival rates, and the 5-year survival was lower than that of patients with UEA [4,6,8].

Given its rarity, the understanding of G-EAC is limited. Previous studies of G-EAC have been largely confined to single-center series or limited sample analyses, with few systematically evaluating treatment outcomes in real-world settings. Further understanding of the clinical and biological characteristics of patients with G-EAC and identification of effective treatment strategies are current medical needs and challenges. Incorporating cases from four tertiary hospitals over a 12-year period, our study constitutes one of the largest multicenter cohorts to date, facilitating a more comprehensive assessment of clinicopathological characteristics, treatment patterns, and prognostic factors. These findings extend prior knowledge by offering real-world evidence on both surgical and systemic therapy strategies for G-EAC.

## Method

### Patient selection

**Figure 1.**
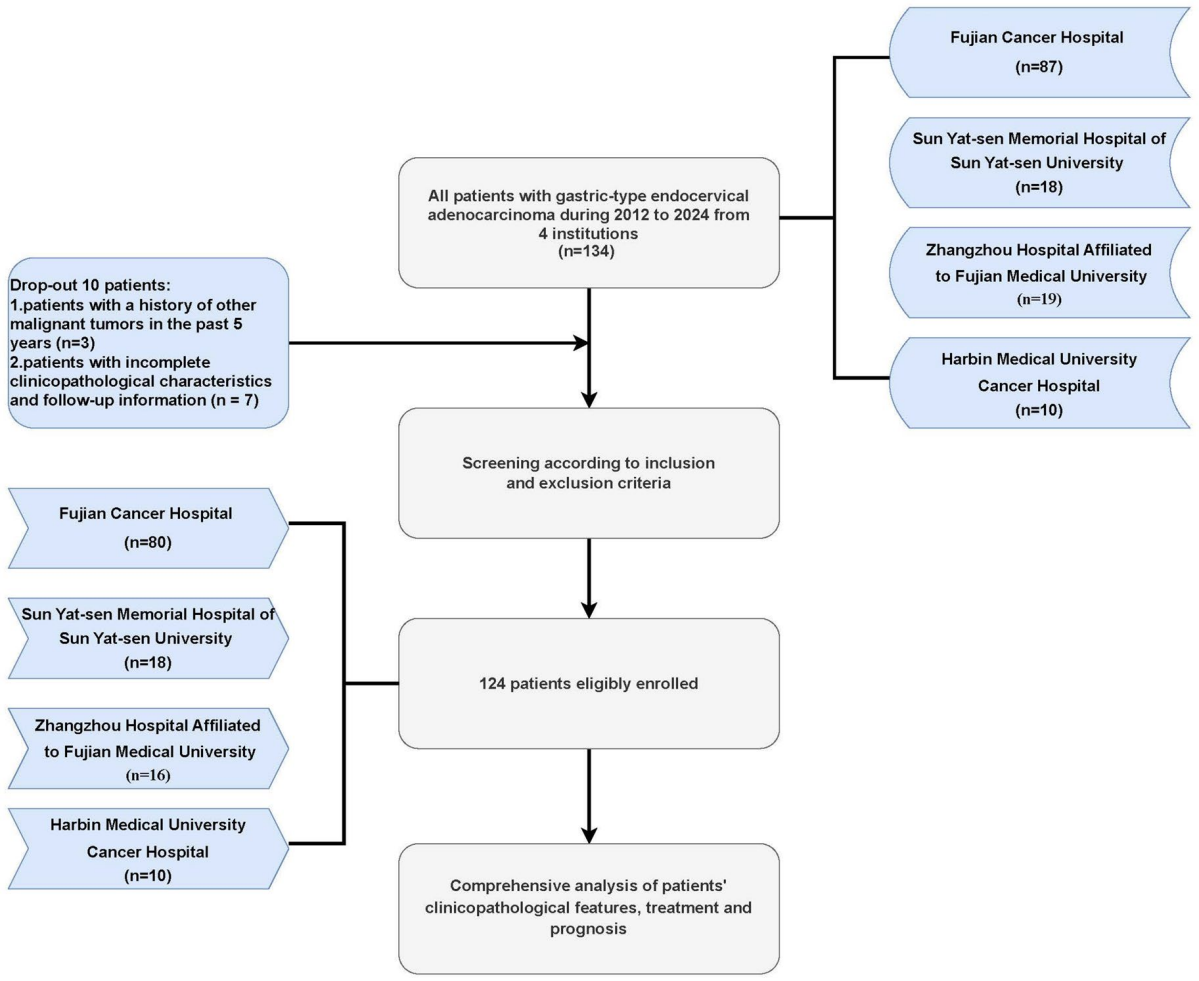
The flowchart of patients’ selection.

The present study included patients with G-EAC diagnosed at four institutions, including Fujian Cancer Hospital (Fuzhou, China), Sun Yat-sen Memorial Hospital of Sun Yat-sen University (Guangzhou, China), Zhangzhou Hospital Affiliated to Fujian Medical University (Zhangzhou, China) and Harbin Medical University Cancer Hospital (Heilongjiang, China), between 1 January 2012, and 28 February 2024. To minimize the potential heterogeneity among hospitals, standardized data collection procedures and uniform inclusion/exclusion criteria were applied across all centers. The specific inclusion criteria were: (i) G-EAC confirmed by pathology; and (ii) patients with complete clinicopathological characteristics and follow-up information. The exclusion criteria were: (i) Patients with a history of other malignant tumors in the past 5 years; and (ii) patients with incomplete clinicopathological characteristics and follow-up information. The flowchart is shown in Figure 1.

Patient data, including basic information, pathological information (including tumor size, differentiation grade and metastasis sites), diagnostic information and treatment regimens, were retrospectively collected using an electronic medical record system.

The clinical stage was determined based on the National Comprehensive Cancer Network (NCCN) Guideline version 1.2022 and the International Federation of Gynecology and Obstetrics (FIGO) Guideline 2018. Regional lymph node metastasis—including pelvic lymph nodes—was categorized as regional metastasis, whereas only para-aortic lymph node involvement and extra-pelvic organ metastasis were classified as distant metastasis. To standardize diagnostic criteria across participating centers, a central pathological review was conducted. All H&E-stained slides were independently re-evaluated by two senior gynecologic pathologists at the coordinating center, and discrepancies were resolved through consensus. All included cases had clear and/or pale eosinophilic cytoplasm with distinct cell borders in a significant portion of the tumor. Tumor differentiation was classified according to the WHO 2020 criteria: well-differentiated tumors exhibited glandular architecture with minimal atypia and could be designated as minimal deviation adenocarcinoma; moderately differentiated tumors displayed partial loss of glandular structures alongside moderate atypia; and poorly differentiated tumors presented solid growth patterns with marked nuclear atypia. The treatments were categorized as chemotherapy, radiotherapy, surgery and combined therapy. Chemotherapy predominantly consisted of platinum–taxane combinations, although a small number of patients received alternative regimens such as fluoropyrimidine–platinum. Radiotherapy typically involved pelvic external beam radiotherapy (EBRT), supplemented in some cases by intracavitary brachytherapy, following institutional protocols. Surgical intervention generally entailed radical hysterectomy and pelvic lymphadenectomy, with cytoreductive surgery reserved for selected advanced cases. The present study was conducted according to the European Society for Medical Oncology-Guidance for Reporting Oncology Real-World Evidence guidelines [9]. Given the retrospective nature of the study and the use of de-identified data, the requirement for informed consent was waived by the ethics committees of all participating institutions.

### Statistical analysis

Data were analyzed using descriptive statistics. Overall survival (OS) was assessed from the time of diagnosis to the time of death by any cause or last follow-up. The last follow-up date for survival analyses was 15 April 2024. Kaplan-Meier survival curves were used to assess the survival of patients. The log-rank test was used to compare the survival curves. The Cox regression model was used for univariate and multivariate survival analysis. *p* < 0.05 (two-tailed) was considered to indicate a statistically significant difference. All statistical analyses were performed using R (version 4.3.2).

## Results

### Patient characteristics

A total of 124 patients diagnosed with G-EAC between 2012 and 2024 were eligible. The median age at diagnosis was 55 years (33–82 years). Considering the high incidence age range of cervical cancer, ages were divided into three stages: <50 years (40/124; 32.3%), 50–64 years (59/124; 47.6%) and >64 years (25/124; 20.2%). A total of 69 (69/124; 55.7%) patients were from rural areas. Patients with G-EAC as the first primary malignancy are up to 113 (113/124; 91.1%). The primary sites of the tumor were categorized as endocervix (19/124; 15.3%), exocervix (12/124; 9.7%), overlapping lesion of cervix uteri (37/124; 29.8%) and cervix uteri (56/124; 45.2%). According to the NCCN staging rules, 42 (42/124; 33.9%) patients had localized tumors, 48 (48/124; 38.7%) patients had regional metastasis, 29 (29/124; 23.4%) patients had distant metastasis, and the metastasis status of 5 (5/124; 4.0%) patients was unknown. Based on the FIGO 2018 staging rules, the clinical stage at presentation was: I (22/124; 17.7%), II (27/124; 21.8%), III (44/124; 35.5%), IV (26/124; 21.0%) or unknown (5/124; 4.0%). The demographics and baseline characteristics of patients included in the present study are summarized in Table 1.

**Table 1.** Demographics and baseline characteristics of patients with G-EAC.

Characteristic	*N* (%)
Year of diagnosis	
2012–2019	36 (29.0%)
2020–2024	88 (71.0%)
Age(years)	
<50	40 (32.2%)
50–64	59 (47.6%)
≥65	25 (20.2%)
Urban/rural	
Rural	69 (55.7%)
Urban	55 (44.3%)
The first primary malignant tumor	
No	11 (8.9%)
Yes	113 (91.1%)
NCCN^a^ stage	
Localized	42 (33.9%)
Regional	48 (38.7%)
Distant	29 (23.4%)
Unknown	5 (4.0%)
The primary site of the tumor	
Endocervix	19 (15.3%)
Exocervix	12 (9.7%)
Overlapping lesion of cervix uteri	37 (29.8%)
Cervix uteri	56 (45.2%)
FIGO^b^ 2018 stage	
I	22 (17.7%)
II	27 (21.8%)
III	44 (35.5%)
IV	26 (21.0%)
Unknown	5 (4.0%)

^a^NCCN: National Comprehensive Cancer Network. ^b^FIGO: International Federation of Gynecology and Obstetrics.

### Pathologic features

A summary of the pathologic features of patients is provided in Table 2. Tumor size (mm) was classified as <20 mm (13/124; 10.5%), 21–39 mm (32/124; 25.8%), ≥40 mm (45/124; 36.3%) or unknown (34/124; 27.4%). Histologically, the tumors were divided into well-differentiated (18/124; 14.5%), moderately differentiated (21/124; 16.9%), poorly differentiated (4/124; 3.2%) and unknown (81/124; 65.3%) groups. Among all 124 patients, 65 (65/124; 52.4%) patients had lymph node metastasis and 32 (32/124; 25.8%) had distant metastasis, including lymph node or organ metastasis. A total of 50 (50/124; 40.3%) patients were found to exhibit uterus involvement, and 29 (29/124; 23.4%), 23 (23/124; 18.6%) and 26 (26/124; 21.0%) patients exhibited involvement of the parietal uterus, vagina and nerve, respectively. In 59 (59/124; 47.6%) cases, lymphovascular invasion was observed.

**Table 2.** Pathologic features of G-EAC.

Characteristic	*N* (%)
Tumor size (mm)	
<20	13 (10.5%)
21–39	32 (25.8%)
≥40	45 (36.3%)
Unknown	34 (27.4%)
Grade	
Well-differentiated	18 (14.5%)
Moderately differentiated	21 (16.9%)
Poorly differentiated	4 (3.2%)
Unknown	81 (65.4%)
Regional lymph node metastasis^a^	
No	59 (47.6%)
Yes	65 (52.4%)
Distant metastasis (Lymph node or organ) (1)	
No	92 (74.2%)
Yes	32 (25.8%)
Involves the uterus	
No	53 (42.7%)
Yes	50 (40.3%)
Unknown	21 (16.9%)
Parauterine involvement	
No	78 (62.9%)
Yes	29 (23.4%)
Unknown	17 (13.7%)
Vaginal invasion	
No	78 (62.9%)
Yes	23 (18.6%)
Unknown	23 (18.6%)
Nerve invasion	
No	68 (54.8%)
Yes	26 (21.0%)
Unknown	30 (24.2%)
Lymphovascular invasion	
No	49 (39.5%)
Yes	59 (47.6%)
Unknown	16 (12.9%)

^a^Regional lymph node metastasis included pelvic lymph node metastasis; distant metastasis referred to para-aortic lymph nodes or distant organ involvement.

### Treatment

Most patients (79/124; 63.7%) began their first treatment within 1 month of tumor discovery. The main features of the treatments are summarized in Table 3. Among 105 (105/124; 84.7%) patients who received chemotherapy, 86 (86/124; 69.4%) patients were treated with paclitaxel combined with platinum, 15 (15/124; 12.1%) were treated with docetaxel combined with platinum, and the remaining patients were treated with oxaliplatin combined with capecitabine. Most of the chemotherapy was performed in three or more cycles, including 55 (55/124; 44.4%) cases with 3–5 cycles and 37 (37/124; 29.8%) cases with six or more cycles. Of the total number of patients, 86 (86/124; 69.3%) patients received radiotherapy and 101 (101/124; 81.5%) patients underwent surgery. Among the patients who received both radiotherapy and surgery, 18 (18/124; 14.5%) were treated with radiotherapy prior to surgery, 51 (51/124; 41.1%) received radiotherapy after surgery, and 18 (18/124; 14.5%) were treated with radiotherapy before and after surgery. Among the cases with systemic therapy and surgery, 18 (18/124; 14.5%) received systemic therapy before surgery, 51 (51/124; 41.1%) received systemic therapy after surgery, and 26 (26/124; 21.0%) received systemic therapy both before and after surgery.

**Table 3.** Treatments of G-EAC.

Characteristic	*N* (%)
The month between diagnosis and first treatment	
0	79 (63.7%)
1	30 (24.2%)
2	6 (4.8%)
3+	9 (7.3%)
Chemotherapy	
No	19 (15.3%)
Yes	105 (84.7%)
Chemotherapy regimens	
Docetaxel combined with platinum	15 (12.1%)
Paclitaxel combined with platinum	86 (69.4%)
Oxaliplatin combined with capecitabine	4 (3.2%)
No chemotherapy	19 (15.3%)
Chemotherapy cycle	
≤2	13 (10.5%)
3–5	55 (44.4%)
≥6	37 (29.8%)
No chemotherapy	19 (15.3%)
Radiotherapy	
No	38 (30.7%)
Yes	86 (69.3%)
Surgery	
No	23 (18.5%)
Yes	101 (81.5%)
Sequence of surgery and radiotherapy	
Radiotherapy prior to surgery	18 (14.5%)
Radiotherapy after surgery	51 (41.1%)
Radiation before and after surgery	18 (14.5%)
No radiotherapy and/or no surgery	37 (29.8%)
Sequence of systemic therapy and surgery	
Systemic therapy before surgery	18 (14.5%)
Systemic therapy after surgery	51 (41.1%)
Systemic therapy both before and after surgery	26 (21.0%)
No systemic therapy and/or surgical procedures	29 (23.4%)

### Survival and prognosis

**Figure 2.**
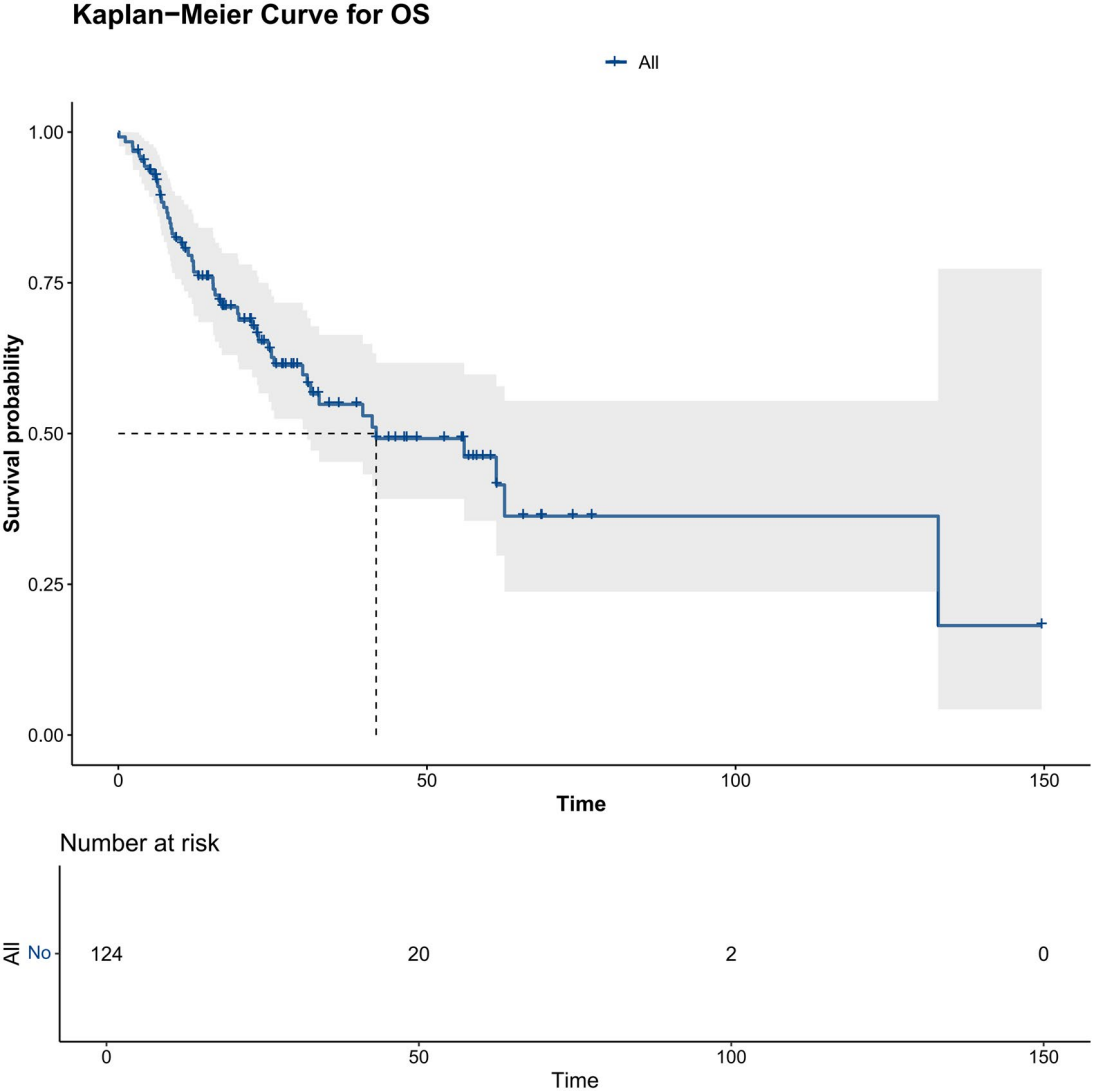
Kaplan-Meier curve of patients with G-EAC illustrates the OS of all the patients included.

**Figure 3.**
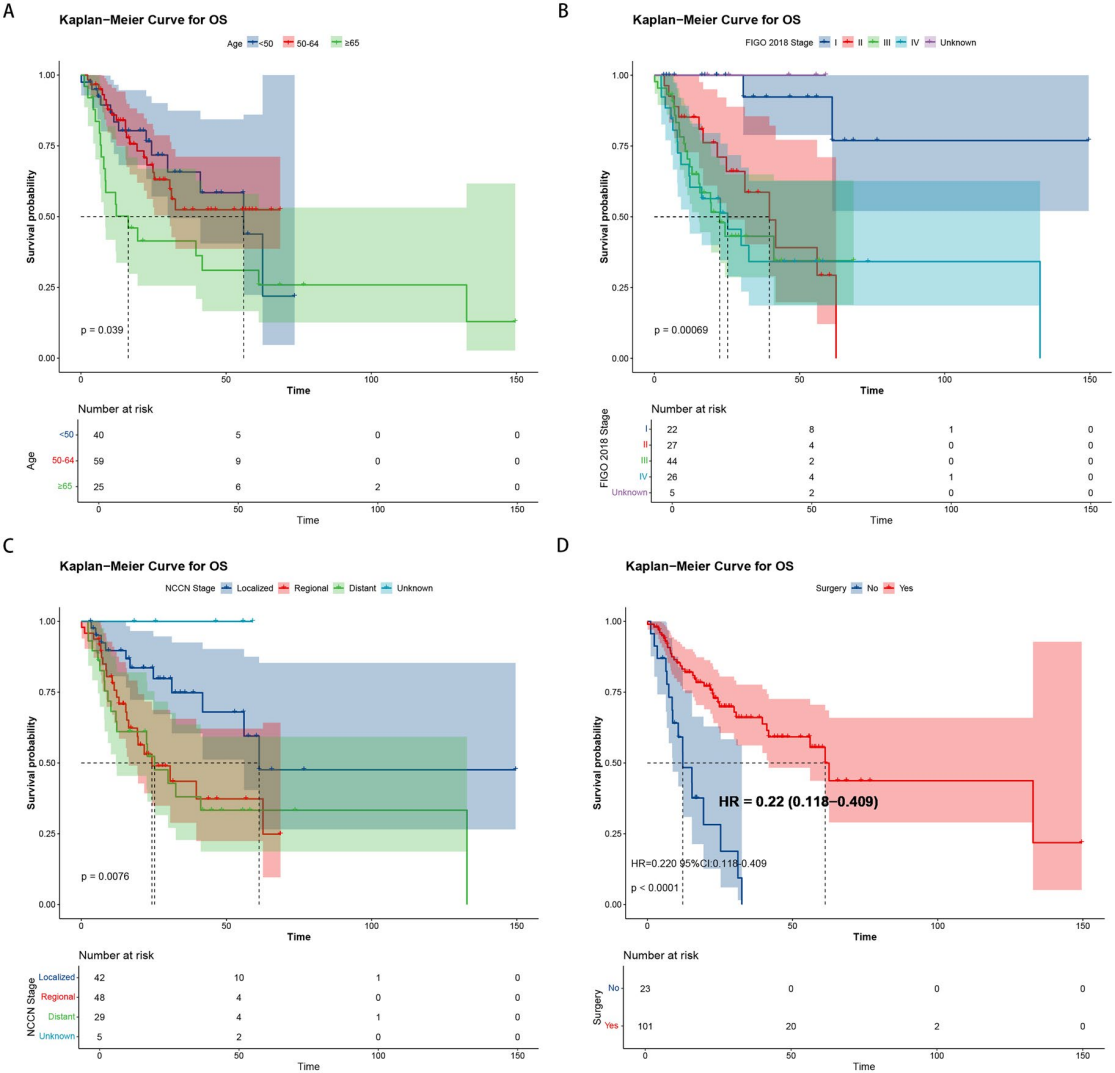
Kaplan–Meier curves for OS in patients with G-EAC according to major clinical characteristics. (A) OS curves stratified by age. (B) OS curves stratified by FIGO 2018 stage. (C) OS curves stratified by NCCN stage. (D) OS curves stratified by surgery.

The median OS time was 41.8 months (30.6 months – not reached). The 1-, 3- and 5-year OS rates were 78.6% (71.5–86.5%), 54.84% (45.3–66.4%) and 46.1% (35.5–59.8%), respectively. The OS curve of 124 patients with G-EAC is shown in Figure 2. In the Kaplan-Meier survival analysis of all cases, differences in OS were observed between groups for age (*p* = 0.039, Figure 3A), FIGO 2018 stage (*p* = 0.00069, Figure 3B), NCCN stage (*p* = 0.0076, Figure 3C), the primary site of the tumor (*p* = 0.022, Figure S1) and surgery (*p* < 0.0001, Figure 3D).

**Figure 4.**
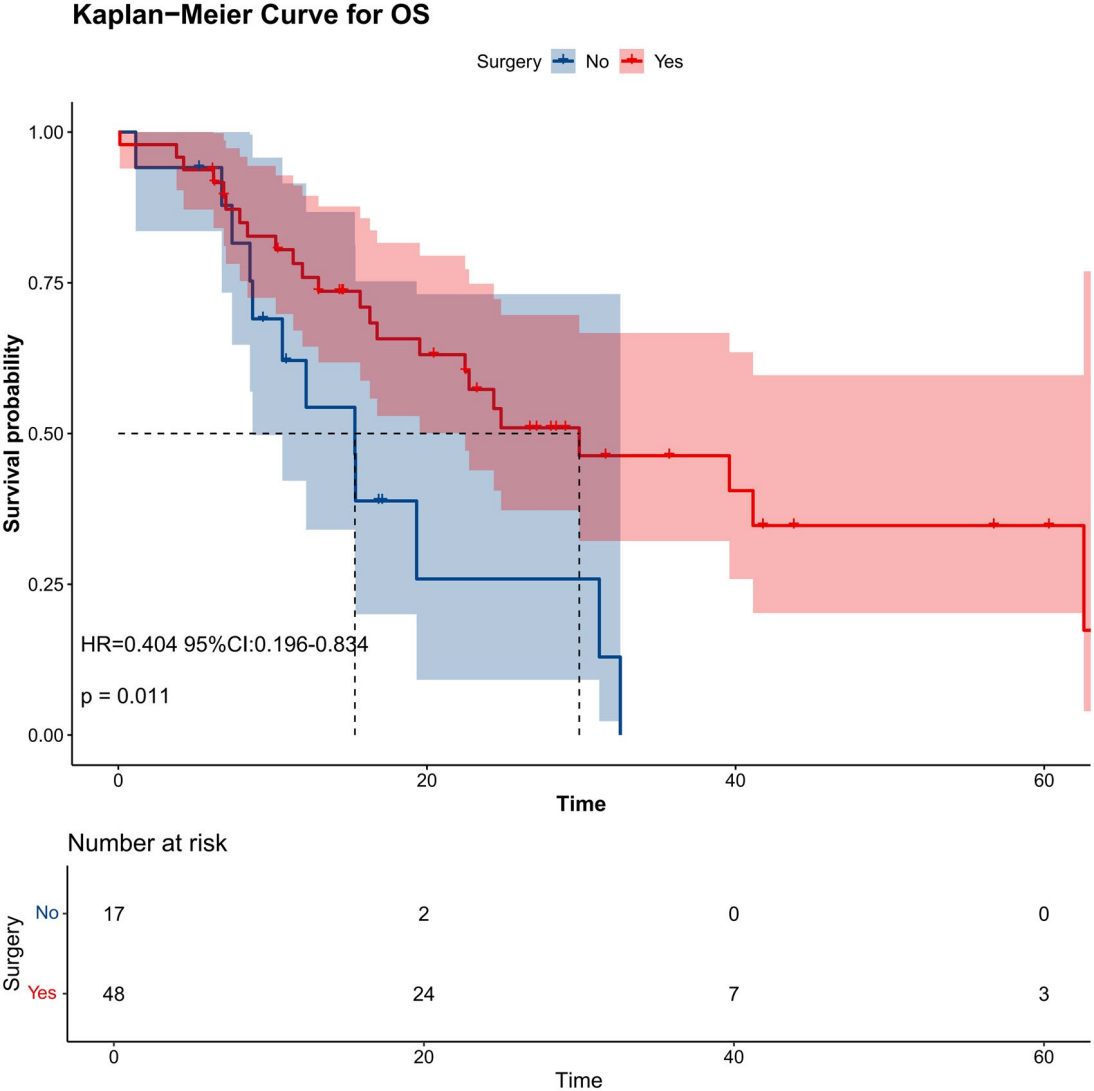
Kaplan-Meier curve of patients with G-EAC in stage IIB-IVA illustrates the OS of advanced patients with or without surgery.

The survival curves of groups based on FIGO 2018 stage showed that the median survival time of patients in stage I was not reached, the median survival time of patients in stage II was 39.6 months (95% CI, 24.8-not reached), the median survival time of patients in stage III was 22.5 months (95% CI, 15.3-not reached) and the median survival time of patients in stage IV was 25.2 months (95% CI, 11.9-not reached). In the Kaplan-Meier survival analysis of patients with stage IIB-IVA, surgical treatment also improved the prognosis of patients compared with that of patients without surgical treatment (*p* = 0.011) (Figure 4).

To further explore the influencing factors of OS in patients with G-EAC, univariate and multivariate Cox regression analysis was performed, and the results are shown in Table 4. The results of univariate analysis showed that age, NCCN stage, FIGO 2018 stage, the time between diagnosis and first treatment, number of chemotherapy cycles, surgery, sequence of surgery and radiotherapy, and sequence of systemic therapy and surgery were potential factors associated with OS (*p* < 0.05).

**Table 4.** Univariate and multivariate analysis of G-EAC prognosis.

	Univariate analysis			Multivariate analysis		
Characteristic	HR^a^	95%CI^b^	*p*-Value	HR	95%CI	*p*-Value
Year of diagnosis						
2012–2019						
2020–2024	1.17	0.62–2.19	0.634			
Age(years)						
<50						
50–64	0.99	0.49–1.99	0.977	0.77	0.28–2.10	0.6089
≥65	2.18	1.05–4.53	0.037	4.71	1.52–14.58	0.0071
Urban/rural						
Rural						
Urban	1.17	0.62–2.19	0.634			
The first primary malignant tumor						
No						
Yes	0.83	0.33–2.09	0.690			
NCCN^c^ stage						
Localized						
Regional	2.39	1.15–4.96	0.019			
Distant	2.68	1.26–5.68	0.010			
Unknown	0.00	0-Inf	0.996			
The primary site of the tumor						
Endocervix						
Exocervix	0.73	0.17–3.1	0.674			
Overlapping lesion of cervix uteri	2.18	0.82–5.77	0.118			
Cervix uteri	0.83	0.31–2.23	0.716			
FIGO^d^ 2018 stage						
I						
II	7.40	1.65–33.16	0.009	15.75	2.86–86.73	0.0015
III	10.12	2.33–43.99	0.002	16.33	2.80–95.03	0.0019
IV	10.06	2.30–43.93	0.002	6.63	1.17–37.74	0.0330
Unknown	0.00	0-Inf	0.996	0.00	0-Inf	0.9979
Tumor size (mm)						
<20						
21–39	2.41	0.67–8.63	0.178			
≥40	3.13	0.93–10.56	0.065			
Unknown	3.48	0.99–12.20	0.051			
Grade						
Well-differentiated						
Moderately differentiated	0.58	0.19–1.74	0.328			
Poorly differentiated	1.15	0.25–5.33	0.862			
Unknown	1	0.48–2.08	0.998			
Regional lymph node metastasis^e^						
No						
Yes	1.67	0.95–2.94	0.075			
Distant metastasis (Lymph node or organ) (5)						
No						
Yes	1.64	0.93–2.9	0.089			
Involves the uterus						
No						
Yes	1.09	0.59–2	0.780			
Unknown	0.96	0.44–2.10	0.925			
Parauterine involvement						
No						
Yes	1.07	0.54–2.09	0.854			
Unknown	1.04	0.49–2.22	0.915			
Vaginal invasion						
No						
Yes	0.90	0.42–1.92	0.786			
Unknown	1.59	0.83–3.04	0.163			
Nerve invasion						
No						
Yes	1.11	0.53–2.32	0.773			
Unknown	1.08	0.57–2.05	0.814			
Vessel carcinoma embolus						
No						
Yes	0.85	0.47–1.54	0.589			
Unknown	1.24	0.53–2.92	0.619			
The month between diagnosis and first treatment						
0						
1	0.36	0.17–0.77	0.009	0.38	0.15–0.96	0.0402
2	0.00	0-Inf	0.996	0.00	0-Inf	0.9967
3+	0.13	0.02–0.96	0.046	0.27	0.03–2.16	0.2168
Chemotherapy						
No						
Yes	1.20	0.56–2.59	0.644			
Chemotherapy regimens						
Docetaxel combined with platinum						
Paclitaxel combined with platinum	0.62	0.30–1.28	0.195			
Oxaliplatin combined with capecitabine	2.51	0.78–8.05	0.122			
No chemotherapy	0.61	0.24–1.57	0.308			
Chemotherapy cycle						
≤2						
3–5	0.14	0.06–0.32	<0.001	0.26	0.09–0.74	0.0119
≥6	0.12	0.05–0.29	<0.001	0.19	0.05–0.70	0.0121
No chemotherapy	0.14	0.05–0.36	<0.001	0.17	0.04–0.74	0.0186
Radiotherapy						
No						
Yes	0.77	0.43–1.40	0.396			
Surgery						
No						
Yes	0.21	0.11–0.39	<0.001	0.10	0.02–0.43	0.0019
Sequence of surgery and radiotherapy						
Radiotherapy prior to surgery						
Radiotherapy after surgery	0.23	0.11–0.50	<0.001	0.23	0.05–1.01	0.0509
Radiotherapy before and after surgery	0.35	0.14–0.88	0.025	0.30	0.06–1.40	0.1242
No radiotherapy and/or no surgery	0.42	0.20–0.90	0.025	1.53	0.41–5.72	0.5305
Sequence of systemic therapy and surgery						
Systemic therapy before surgery						
Systemic therapy after surgery	0.22	0.10–0.45	<0.001	13.45	1.62–111.37	0.0160
Systemic therapy both before and after surgery	0.34	0.15–0.77	0.009	23.00	2.53–208.68	0.0053
No systemic therapy and/or surgical procedures	0.24	0.10–0.57	0.001	5.56	0.82–37.79	0.0794

^a^HR: hazard ratio. ^b^CI: Confidence interval. ^c^NCCN: National Comprehensive Cancer Network. ^d^FIGO: International Federation of Gynecology and Obstetrics. ^e^Regional lymph node metastasis included pelvic lymph node metastasis; distant metastasis referred to para-aortic lymph nodes or distant organ involvement.

**Figure 5.**
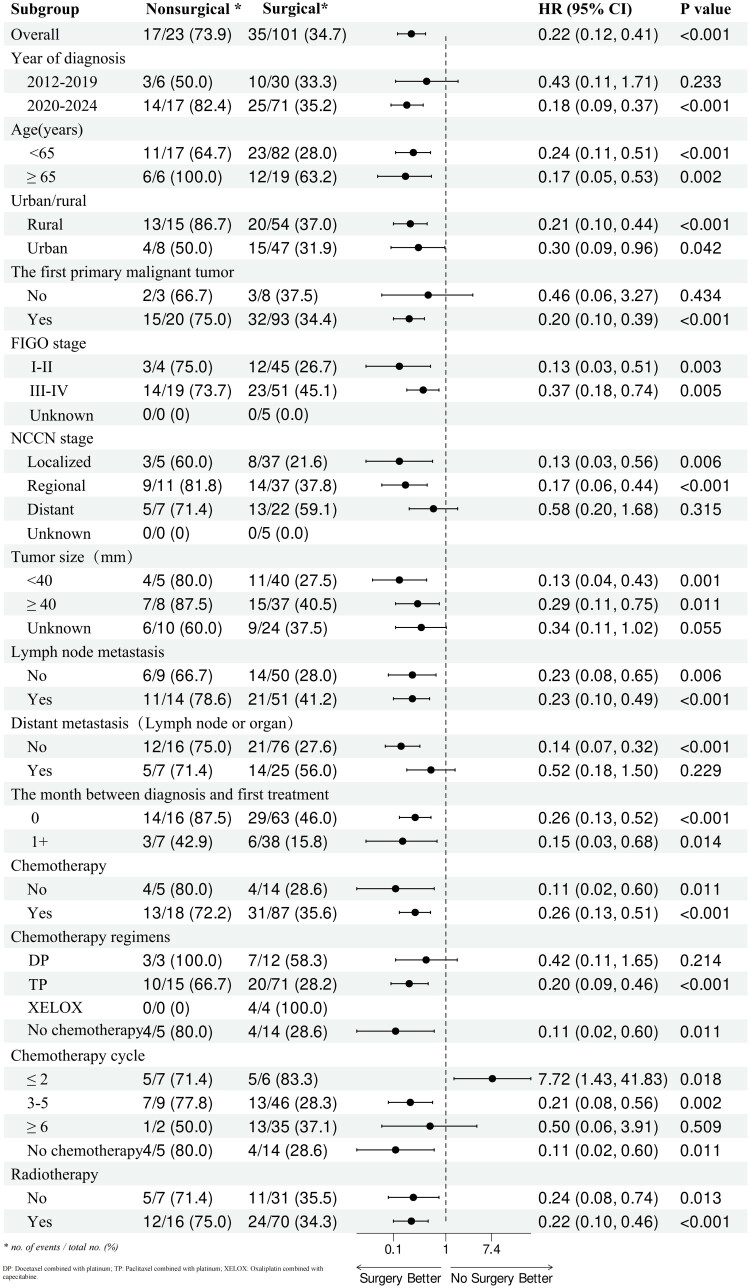
Forest plot of subgroup analysis according to surgical status.

The significant factors identified in the univariate analysis were further included in the multivariate Cox regression model using a backward stepwise selection method. Age ≥65 years [hazard ratio (HR), 4.71; 95% CI, 1.52–14.58; *p* = 0.0071], FIGO 2018 stage II (HR, 15.75; 95% CI, 2.86–86.73; *p* = 0.0015), stage III (HR, 16.33; 95% CI, 2.80–95.03; *p* = 0.0019), stage IV (HR, 6.63; 95% CI, 1.17–37.74; *p* = 0.0330), systemic therapy after surgery (HR, 13.45; 95% CI, 1.62–111.37; *p* = 0.0160), and systemic therapy both before and after surgery (HR, 23; 95% CI, 2.53–208.68; *p* = 0.0053) were identified as independent factors influencing OS. By contrast, an interval of 1 month between diagnosis and first treatment (HR, 0.38; 95% CI, 0.15–0.96; *p* = 0.0402), 3–5 cycles of chemotherapy (HR, 0.26; 95% CI, 0.09–0.74; *p* = 0.0119), ≥6 cycles of chemotherapy (HR, 0.19; 95% CI, 0.05–0.70; *p* = 0.0121), and surgery (HR, 0.10; 95% CI, 0.02–0.43; *p* = 0.0019) were determined to be independent protective factors for OS. Subgroup analysis of surgical patients showed that patients with localized and regional G-EAC could benefit from surgery. The specific results are shown in Figure 5.

## Discussion

G-EAC is a rare mucinous adenocarcinoma of the uterine cervix. It is characterized by high-risk HPV-negativity, atypical symptoms and signs, complex and variable pathohistological features, and immunophenotypes [8,10]. Based on the difficulty of diagnosis, the incidence rate might be underestimated, and there is a higher proportion of late-stage patients at first diagnosis. The biological behavior of G-EAC is highly malignant, invasive, metastatic and drug-resistant, and its prognosis is poor [8]. As originally reported by Kojima et al. gastric immunophenotyping was associated with lower 5-year disease-specific survival and an elevated risk of disease recurrence [4]. This evidence was reaffirmed by multiple previous studies [8,11]. The present study also showed that the 1-, 3- and 5-year OS rates of G-EAC were 79.3, 55.3 and 46.5%, significantly lower than UEA. Patients with stage I disease had an improved OS rate compared with patients with other stages (Figure 3B; *p* = 0.00069).

G-EAC commonly exhibits local invasion, including the full periphery of the cervical canal, parietal uterus, uterus, lymph-vascular space and nerve [12]. Furthermore, it is highly aggressive and susceptible to metastasis, such as ovarian, lymph node, other organs in the pelvic and abdominal cavities, greater omentum, and peritoneal dissemination metastases [6]. The present study demonstrated that metastasis and invasion were common in patients with G-EAC, with 52.4% (65/124) exhibiting lymph node metastasis, 47.6% (59/124) exhibiting lymphovascular invasion and 40.3% (50/124) exhibiting involvement of the uterus. However, the proportions of distant metastasis (lymph node or organ) and nerve invasion were only 25.8% (32/124) and 21.0% (26/124), respectively. This may be related to the fact that the patients in the present study accounted for a large proportion of early-stage patients.

The diagnosis of G-EAC is primarily based on histopathology. Characteristic features include tumor cells with abundant clear or pale eosinophilic cytoplasm, distinct cell borders, and frequent apical mucin [13]. However, the disease exhibits heterogeneous histological patterns, which contribute to the diagnostic difficulty [14]. Immunohistochemical (IHC) staining can serve as an adjunct when morphology is inconclusive [15,16]. Typical immunophenotypic findings of G-EAC include positivity for gastric markers such as MUC6 and HIK1083, as well as frequent p53 aberrant expression, while p16 usually shows negative or only focal/patchy staining. In addition, carbonic anhydrase IX may be positive in some cases [17–19]. Previous study has also demonstrated the importance of using large tumor area sections for HER2 testing in G-EAC cases [20]. Wang et al. considered HER2 as a poor prognostic marker for G-EAC [21]. However, the appropriate HER2 IHC cut-off in G-EAC should be further explored in large-cohort prospective studies. These features help distinguish G-EAC from HPV-associated UEA.

The results of the present multivariate analysis showed that no chemotherapy was an independent protective prognostic factor. This may be related to the fact that G-EAC is insensitive to chemotherapy and is easily resistant to chemotherapy [22]. Conversely, surgery constituted an independent favorable prognostic factor, reinforcing its role as a first-line intervention for G-EAC patients [21]. However, further prospective studies are needed to verify whether cytoreductive surgery can improve survival in patients in the advanced stages. It should be noted that the finding that systemic therapy administered after surgery (HR 13.45) or both before and after surgery (HR 23.00) was associated with worse OS is most likely due to confounding by indication. Patients who received systemic therapy in these sequences generally had more advanced disease, higher tumor burden, or poorer baseline conditions, which inherently predicted a worse prognosis. Therefore, this association should not be interpreted as evidence that systemic therapy worsens survival, but rather as a reflection of underlying disease severity and treatment selection bias.

In addition, genomic study of G-EAC have shown that the most common mutated gene is TP53, followed by KRAS, SMAD4, lysine methyltransferase 2D, erb-b2 receptor tyrosine kinase 3 and ring finger protein 43, similar to gastrointestinal adenocarcinoma [23]. Recent molecular reviews have reinforced these observations, highlighting recurrent mutations in TP53, KRAS, and other pathways as hallmarks of HPV-independent cervical adenocarcinomas [10,24,25]. Park et al. also found that there were a large number of mutations in epithelial-mesenchymal transition (EMT)-related genes in G-EAC, suggesting that the EMT-related pathway may serve a role in tumor dissemination and chemotherapy resistance in G-EAC [5]. Based on this, the genetic characteristics of G-EAC will be further explored in the future to reveal the biological mechanisms of G-EAC, leading to the development of more robust practice guidelines in the future.

The present study investigated the real-world clinicopathological features and treatment outcomes of G-EAC. Compared with prior reports that primarily emphasized descriptive pathology or single-institution experiences, this study offered new real-world evidence derived from a multicenter cohort, underscoring the prognostic importance of surgery and the restricted utility of chemotherapy. These results corroborated earlier observations of poor outcomes in G-EAC and further expanded the current literature by elucidating treatment-related prognostic factors within a larger and more diverse patient population.

However, as this was a retrospective analysis, the limitations, including loss of information and inherent biases, should not be ignored. First, as a retrospective analysis, some clinicopathological details, including immunohistochemical results and molecular profiling, were not available. Second, although central pathological review was conducted, inter-hospital heterogeneity in surgical techniques, adjuvant therapies, and clinical management could not be fully eliminated. Third, detailed treatment parameters such as radiotherapy dose, chemotherapy regimens beyond platinum–taxane, and surgical procedures were inconsistently documented across centers, which may limit the clinical granularity of our findings. Finally, treatment selection was not randomized, and the observed prognostic effect of systemic therapy sequences may reflect confounding by indication. Therefore, further large-scale, prospective, and ideally randomized studies are needed to confirm these results.

## Conclusion

In conclusion, this multicenter retrospective study highlights the distinct clinicopathological features and poor prognosis of G-EAC. Our results reinforce that surgery remains the cornerstone of treatment, while chemotherapy appears to provide limited benefit in this population. The findings underscore the need for prospective, ideally randomized, clinical trials to validate the role of cytoreductive surgery, optimize systemic therapy strategies, and ultimately improve outcomes for patients with this aggressive, HPV-independent cervical adenocarcinoma subtype.

## Supplementary Material

Supplemental Material

Figure S1.tif

## Data Availability

Data for this study can be obtained by emailing the corresponding author. Code and other materials available on request from the authors.
