# Supplementary material for: Comprehensive clinical analysis of gastric-type endocervical adenocarcinoma: a real-world multicenter study
Source: Ann Med. 2025 Nov 14;57(1):2584735. doi: 10.1080/07853890.2025.2584735 (PMC12621345; doi:10.1080/07853890.2025.2584735)
Supplement: Supplemental Material [file IANN_A_2584735_SM5398.docx]

Figure S1: Kaplan–Meier curves for OS in patients with G-EAC according to additional subgroups. (A) OS curves stratified by tumor size. (B) OS curves stratified by primary tumor site. (C) OS curves stratified by chemotherapy. (D) OS curves stratified by radiotherapy.
