# Supplementary figures and images for: Comprehensive clinical analysis of gastric-type endocervical adenocarcinoma: a real-world multicenter study
Source: Ann Med. 2025 Nov 14;57(1):2584735. doi: 10.1080/07853890.2025.2584735 (PMC12621345; doi:10.1080/07853890.2025.2584735)

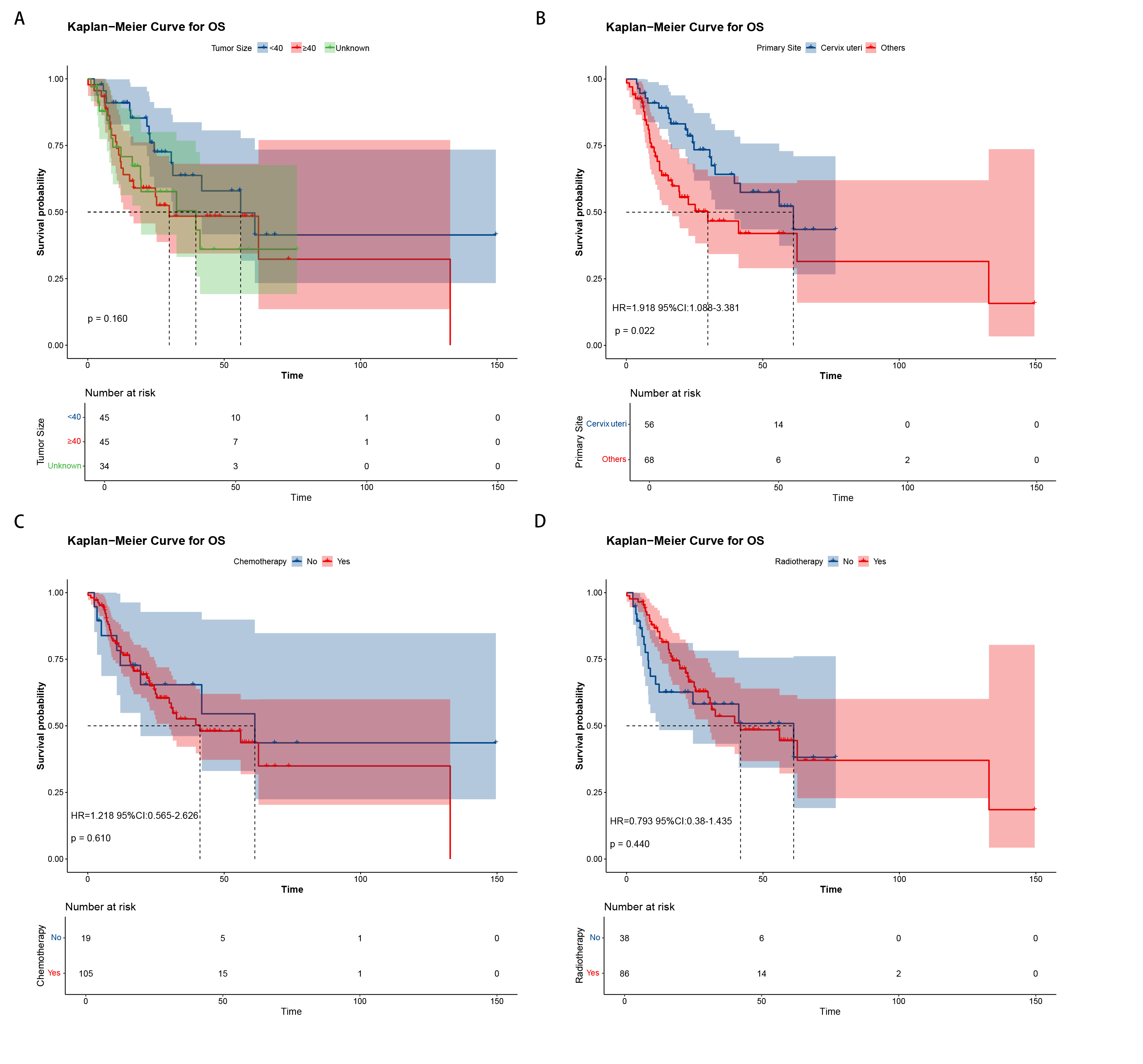

Supplement: Figure S1.tif [file IANN_A_2584735_SM6104.tif]
